# Taxon-Specific Physiological and Rhizosphere Responses of Deciduous Ornamental Shrubs to Humic- and Fulvic Acid-Based Biostimulant Treatment

**DOI:** 10.3390/plants15101455

**Published:** 2026-05-10

**Authors:** Dezső Kovács, Katalin Horotán, László Orlóci, Katalin Juhos, István Dániel Mosonyi, Zsanett Istvánfi, Magdolna Sütöri-Diószegi, Szilvia Kisvarga

**Affiliations:** 1 Institute of Landscape Architecture, Urban Planning and Garden Art, Hungarian University of Agriculture and Life Sciences (MATE), 1223 Budapest, Hungary; kovacsdezso.zsztgy@gmail.com; 2 Institute of Biology, Eszterházy Károly Catholic University, 3300 Eger, Hungary; 3 Ornamental Plant and Green System Management Research Group, Institute of Landscape Architecture, Urban Planning and Garden Art, Hungarian University of Agriculture and Life Sciences (MATE), 1223 Budapest, Hungary; orloci.laszlo@uni-mate.hu (L.O.); istvanfi.zsanett@uni-mate.hu (Z.I.); kisvarga.szilvia@uni-mate.hu (S.K.); 4 Doctoral School of Plant Sciences, Hungarian University of Agriculture and Life Sciences (MATE), 1223 Budapest, Hungary; 5 Department of Agro-Environmental Studies, Hungarian University of Agriculture and Life Sciences, 1117 Budapest, Hungary; juhos.katalin@uni-mate.hu; 6 Department of Floriculture and Dendrology, Institute of Landscape Architecture, Urban Planning and Garden Art, Hungarian University of Agriculture and Life Sciences (MATE), 1223 Budapest, Hungary; mosonyi.istvan.daniel@uni-mate.hu (I.D.M.); sutorine.dioszegi.magdolna@uni-mate.hu (M.S.-D.)

**Keywords:** biostimulant, BiStep, ornamental shrubs, net photosynthesis, transpiration, stomatal density, chlorophyll index

## Abstract

In containerized finished plant production, the effects of biostimulants in nursery practice are often judged primarily on the basis of visual condition, while a more precise interpretation of treatment response requires leaf-level physiological and rhizosphere-level indicators. The aim of our study was to determine how the humic- and fulvic acid-based BiStep biostimulant influences the physiological functioning and, in part, the rhizosphere enzyme activity of three deciduous ornamental shrub taxa widely used both in nursery finished plant production and in urban green space plantings, namely, *Forsythia* × *intermedia* ‘Beatrix Farrand’, *Weigela florida* ‘Eva Rathke’, and *Viburnum opulus* ‘Roseum’, under commercial container conditions. In the experiment, control and biostimulant treatments were compared. Treatment effects were evaluated on the basis of net photosynthesis (Pn); transpiration (E); chlorophyll content; stomatal density; stomatal length; and acid phosphatase (ACP), alkaline phosphatase (ALP), and β-glucosidase (GLUC) activities. For Pn, a significant taxon × treatment interaction was observed (*p* = 0.002). Pn showed taxon-dependent numerical changes under BiStep: values were 22.212 µmol CO_2_ m^−2^ s^−1^ in *F. intermedia*, 4.182 µmol CO_2_ m^−2^ s^−1^ in *W. florida*, and 3.370 µmol CO_2_ m^−2^ s^−1^ in *V. opulus*, but pairwise differences from the control were not statistically significant. Transpiration also showed a significant taxon × treatment interaction (*p* < 0.001), although BiStep–control differences were not significant within taxa. Stomatal density increased significantly in *F. intermedia* and *W. florida*, while the BiStep–control difference was not significant in *V. opulus*. Chlorophyll content increased only in *W. florida* (from 699.6 to 924.4 µg g^−1^ fresh weight), but this change was not statistically significant. ACP activity showed significant treatment and interaction effects (*p* = 0.0107; *p* = 0.00546), whereas ALP and GLUC did not show a consistent treatment response. Based on the results, the effect of BiStep was clearly taxon-dependent and functionally selective. Therefore, in nursery finished plant production and subsequent urban plant use, it should not be considered a universally effective input, but rather a biostimulant whose relevance depends on the specific physiological and rhizosphere-level response of the taxon.

## 1. Introduction

In containerized ornamental shrub production, the practical evaluation of biostimulants often relies on the visual condition of plants [1,2], while the essential and detectable effects of treatments can be captured in leaf-level physiological functioning [3,4,5]. Net photosynthesis (Pn) describes the current intensity of carbon uptake, whereas transpiration (E) reflects the instantaneous burden of water flux and the functional status of stomatal regulation [6,7]. In a container system, these indicators are particularly sensitive because root zone volume is limited, the water-holding capacity and nutrient supply capacity of the substrate can change rapidly [8], and the microclimate of the canopy zone can substantially modify stomatal status over a short period [1,9]. Against this background, the effect of biostimulants can be interpreted reliably only if the analysis examines treatment-related shifts [10] and treats the fundamental differences among taxa as an independent factor [11,12].

The leaf-level effects of biostimulants can only be interpreted reliably through the combined examination of several functionally related parameters [13]. Gas exchange parameters reflect the current intensity of plant functioning, but their variation alone does not separate the components arising from structural characteristics, physiological regulation, and the current environmental state [14,15]. Stomatal density and stomatal length therefore provide important morphological background for interpreting net photosynthesis and transpiration, since they define the basis of the leaf’s diffusive capacity [16,17]. At the same time, the chlorophyll index provides additional information on the status of the light-converting system of the photosynthetic apparatus, thus helping to assess whether treatment-related shifts appear merely at the level of regulation or also affect a broader range of the leaf’s functional organization [18,19]. This is particularly important in the case of biostimulants, since their effect is usually not exerted through a single physiological pathway, but through the coordinated modification of water balance, carbon assimilation, and stress response [20,21]. The combined evaluation of gas exchange, chlorophyll index, and stomatal parameters is therefore better suited to describing taxon-specific responses than the separate analysis of any one indicator alone [22].

The effects of humic- and fulvic acid-based biostimulants can be interpreted through several interrelated processes [21]. These substances may alter the functional status of the root zone and the solubility and availability of nutrients [23], which is especially important in container cultivation, where water and nutrient turnover in the substrate changes rapidly within a confined space [24,25]. Owing to the greater mobility of fulvic acid fractions, the effect of these products may also appear at more sensitive levels of plant regulation, especially where changes in root environment status affect stomatal functioning and gas exchange within a short time [26,27]. The practical significance of these products can therefore be captured primarily in how they modify the balance between carbon assimilation and water loss, which is most directly indicated by net photosynthesis and transpiration [28,29]. Stomatal parameters and the chlorophyll index complement this interpretation with additional structural and functional relationships [30,31].

Responses induced by biostimulants in ornamental shrubs are presumably taxon-dependent, because the individual taxa do not respond to treatment from the same anatomical and physiological baseline [32]. Leaf structural features, the characteristics of the stomatal apparatus, and the dynamics of regulation together determine the direction and magnitude of the shift caused by the same product in gas exchange [21,33]. Accordingly, the relationship among Pn, E, and stomatal parameters cannot be interpreted according to the same logic in every taxon [13]. The taxon-specific approach is therefore not merely a more detailed analysis, but a condition for the validity of the conclusions that can be drawn [34].

A previous study on *W. florida* ‘Eva Rathke’ evaluated morphological and physiological responses to several biostimulant and growth-promoter products, including BiStep, KelPak, and Yeald Plus. The present work differs from that study in three main aspects: it focuses specifically on BiStep; extends the evaluation from a single taxon to three deciduous ornamental shrub taxa; and integrates leaf-level gas exchange, chlorophyll content, stomatal traits, and rhizosphere enzyme activities within a taxon-comparative framework. The aim of the present study was to determine whether the humic- and fulvic acid-based BiStep biostimulant induces comparable or taxon-specific physiological and rhizosphere-level responses in three deciduous ornamental shrub taxa: *Forsythia × intermedia* ‘Beatrix Farrand’, *Weigela florida* ‘Eva Rathke’, and *Viburnum opulus* ‘Roseum’. The selected taxa are ornamental shrubs widely used in nursery production and also important in urban green space applications, so the interpretation of treatment responses is justified from a practical application standpoint as well. Relative to the control, treatment effects were examined through the combined evaluation of net photosynthesis (Pn), transpiration (E), chlorophyll content, stomatal density, and stomatal length. We hypothesized that BiStep would induce detectable shifts in leaf-level functioning, but that their magnitude and direction would differ among taxa; therefore, treatment responses can only be interpreted reliably within a taxon-specific framework.

## 2. Results

### 2.1. Leaf-Level Gas Exchange

**Figure 1:**
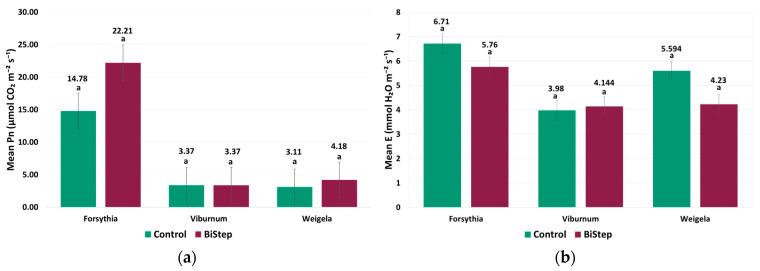
Leaf-level gas exchange responses of the three ornamental shrub taxa under control and BiStep treatments. (**a**) Net photosynthesis (Pn, µmol CO_2_ m^−2^ s^−1^); (**b**) Transpiration (E, mmol H_2_O m^−2^ s^−1^). Bars represent mean values ± SE for each treatment within each taxon. Different letters indicate significant differences among treatments within a given taxon (*p* < 0.05).

The pattern of net photosynthesis (Pn) was strongly determined by taxon (*p* < 0.001), while the main effect of treatment was not significant across the full dataset (*p* = 0.357) (Figure 1a). At the same time, the interaction between taxon and treatment was significant (*p* = 0.002), indicating a taxon-dependent pattern. In *F. intermedia*, mean Pn was 22.212 µmol CO_2_ m^−2^ s^−1^ under BiStep treatment, compared with 14.782 µmol CO_2_ m^−2^ s^−1^ in the control, although this difference was not statistically significant (*p* > 0.05). In *W. florida*, Pn was 4.182 µmol CO_2_ m^−2^ s^−1^ under BiStep and 3.110 µmol CO_2_ m^−2^ s^−1^ in the control group, although this difference was not statistically significant (*p* > 0.05). In *V. opulus*, mean Pn values remained practically identical between BiStep and the control (3.370 µmol CO_2_ m^−2^ s^−1^ in both groups).

For transpiration (E), taxon (*p* = 0.018), treatment (*p* < 0.001), and the taxon × treatment interaction (*p* < 0.001) were all significant, suggesting that overall response patterns differed among taxa and treatments (Figure 1b). In *F. intermedia*, transpiration under BiStep was 5.760 mmol H_2_O m^−2^ s^−1^, while the control value was 6.710 mmol H_2_O m^−2^ s^−1^, representing a non-significant difference of −0.950 mmol H_2_O m^−2^ s^−1^ (*p* > 0.05). In *W. florida*, E was 4.230 mmol H_2_O m^−2^ s^−1^ under BiStep and 5.594 mmol H_2_O m^−2^ s^−1^ in the control group, representing a non-significant difference of −1.364 mmol H_2_O m^−2^ s^−1^ (*p* > 0.05). In *V. opulus*, E was numerically higher under BiStep (4.144 mmol H_2_O m^−2^ s^−1^) than in the control (3.980 mmol H_2_O m^−2^ s^−1^), representing a non-significant difference of 0.164 mmol H_2_O m^−2^ s^−1^.

### 2.2. Chlorophyll Index

**Figure 2:**
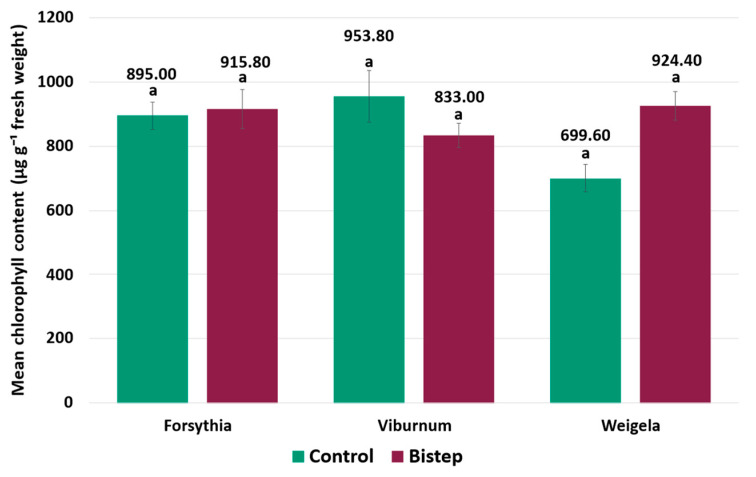
Chlorophyll content of the three ornamental shrub taxa under control and BiStep treatments. Bars represent mean values ± SE for each treatment within each taxon. Chlorophyll content is expressed as µg g^−1^ fresh weight. Different letters indicate significant differences among treatments within a given taxon (*p* < 0.05).

Chlorophyll content was evaluated within each taxon by comparing the control and BiStep treatments. The treatment effect was not statistically significant between the control and BiStep treatments in any of the examined taxa (*p* > 0.05 in all cases) (Figure 2).

In *W. florida*, chlorophyll content was numerically higher under BiStep treatment, increasing from 699.6 to 924.4 µg g^−1^ fresh weight, although this difference was not statistically significant. In *F. intermedia*, chlorophyll content was 915.8 µg g^−1^ fresh weight in the BiStep group and 895.0 µg g^−1^ fresh weight in the control group, representing a non-significant difference (*p* > 0.05). In *V. opulus*, lower chlorophyll content was measured under BiStep, at 833.0 µg g^−1^ fresh weight, compared with 953.8 µg g^−1^ fresh weight in the control group, also representing a non-significant difference (*p* > 0.05).

### 2.3. Stomatal Parameters

**Figure 3:**
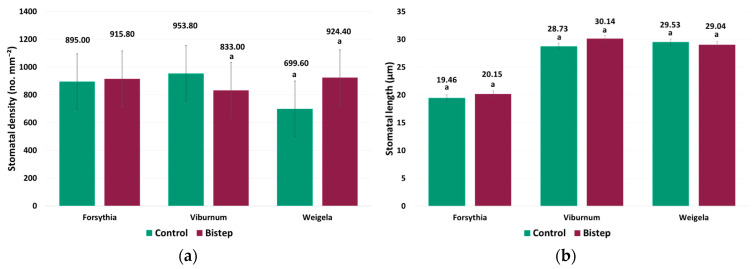
Stomatal traits of the three ornamental shrub taxa under control and BiStep treatments. (**a**) Stomatal density (no. mm^−2^); (**b**) Stomatal length (µm). Bars represent mean values ± SE for each treatment within each taxon. Different letters indicate statistically significant differences among treatments within a given taxon (*p* < 0.05).

For stomatal density, the effects of taxon (*p* < 0.001), treatment (*p* < 0.001), and the taxon × treatment interaction (*p* < 0.001) were significant, clearly indicating a taxon-dependent treatment response (Figure 3a). In *F. intermedia*, stomatal density increased from 552.80 to 599.83 no. mm^−2^ under BiStep, representing a significant increase (*p* = 0.020). In *W. florida*, stomatal density increased from 299.07 to 352.17 no. mm^−2^, representing a significant increase of 53.10 no. mm^−2^ (*p* = 0.006). In *V. opulus*, the difference remained much smaller: stomatal density was 423.50 no. mm^−2^ under BiStep and 411.87 no. mm^−2^ in the control, representing a non-significant difference (*p* > 0.05).

A linear mixed model was applied to evaluate stomatal length because data obtained from several measurement points within a leaf constituted repeated observations. Among the fixed effects, taxon (*p* < 0.001), treatment (*p* = 0.040), and the taxon × treatment interaction (*p* < 0.001) were also significant, indicating that the effect of treatment was expressed differently among taxa (Figure 3b). In *F. intermedia*, stomatal length increased from 19.456 to 20.148 µm; however, this difference was not statistically significant (*p* > 0.05). In *V. opulus*, stomatal length was 30.144 µm under BiStep and 28.732 µm in the control, but this difference was not statistically significant (*p* > 0.05). In contrast, stomatal length did not differ significantly between the control and BiStep treatments in *W. florida*.

### 2.4. Rhizosphere Soil Enzyme Activities

Based on the statistical evaluation of rhizosphere enzyme activities, the effect of treatment was significant for ACP (*p* = 0.0107), and the taxon × treatment interaction also showed a significant difference (*p* = 0.00546), indicating a taxon-dependent treatment response. For ALP, only taxon had a significant effect (*p* = 0.0227), while treatment (*p* = 0.1879) and the taxon × treatment interaction (*p* = 0.2240) were not significant. For GLUC, neither taxon (*p* = 0.199), treatment (*p* = 0.422), nor the taxon × treatment interaction (*p* = 0.505) had a significant effect.

**Figure 4:**
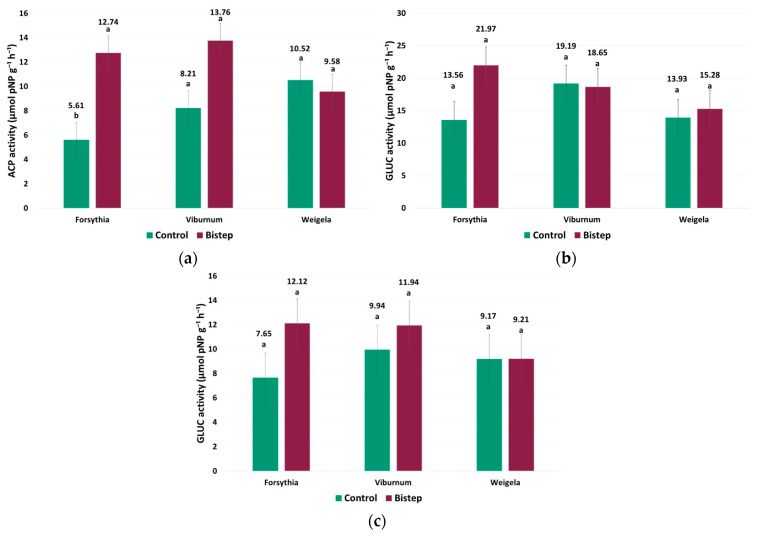
Rhizosphere soil enzyme activities of the three ornamental shrub taxa under control and BiStep treatments. (**a**) Acid phosphatase (ACP) activity; (**b**) Alkaline phosphatase (ALP) activity; (**c**) β-glucosidase (GLUC) activity. Enzyme activities are expressed as µmol pNP g^−1^ h^−1^. Bars represent mean values ± SE for each treatment within each taxon. Different letters indicate statistically significant differences among treatments within a given taxon (*p* < 0.05).

ACP activity showed a taxon-dependent response to BiStep: it increased from 5.610 to 12.743 μmol pNP g^−1^ h^−1^ in *F. intermedia* (*p* = 0.006); in *V. opulus*, it was numerically higher under BiStep (13.756) than in the control (8.208), but this difference was not statistically significant (*p* = 0.053); and it decreased from 10.519 to 9.585 μmol pNP g^−1^ h^−1^ in *W. florida* with no significant difference between treatments. This pattern is consistent with the significant interaction detected for ACP (Figure 4a).

ALP activity showed different baseline levels among taxa, and neither treatment (*p* = 0.188) nor the taxon × treatment interaction (*p* = 0.224) had a significant effect. Control and BiStep values were 13.560 and 21.970 μmol pNP g^−1^ h^−1^ in *F. intermedia*, 19.188 and 18.652 μmol pNP g^−1^ h^−1^ in *V. opulus*, and 13.933 and 15.275 μmol pNP g^−1^ h^−1^ in *W. florida*; however, these differences were not statistically significant within any taxon (Figure 4b).

No consistent treatment pattern emerged for GLUC activity (Figure 4c), and neither taxon (*p* = 0.199), treatment (*p* = 0.422), nor the taxon × treatment interaction (*p* = 0.505) had a significant effect. In *F. intermedia*, GLUC activity was numerically higher under BiStep (12.124 μmol pNP g^−1^ h^−1^) than in the control (7.647 μmol pNP g^−1^ h^−1^), although this difference was not statistically significant. In *V. opulus*, GLUC activity was also slightly higher under BiStep (11.940 μmol pNP g^−1^ h^−1^) compared with the control (9.944 μmol pNP g^−1^ h^−1^), without a significant difference. In *W. florida*, values were nearly identical between treatments (9.205 vs. 9.167 μmol pNP g^−1^ h^−1^), indicating no treatment effect.

## 3. Discussion

The present study extends previous work on biostimulant responses in *W. florida* by shifting the focus from the comparative evaluation of several commercial products in a single taxon to the taxon-specific interpretation of BiStep responses across three deciduous ornamental shrub taxa. This change in scope is important because the results demonstrate that the physiological and rhizosphere-level effects of BiStep cannot be generalized across taxa. Based on the responses outlined by leaf-level gas exchange, chlorophyll content, stomatal morphological traits, and certain rhizosphere enzyme activities, the magnitude and interpretation of the treatment effect appear to be closely related to the physiological characteristics of the taxon.

### 3.1. Taxon-Dependent Leaf-Level Responses and Their Interpretation

This was supported by the significant taxon × treatment interaction detected for net photosynthesis and transpiration: in *F. intermedia*, the increase in Pn was associated with a decrease in E; in *W. florida*, the same direction appeared at lower absolute values; however, in *V. opulus*, Pn remained unchanged and E increased slightly. These contrasting response patterns are consistent with Antonucci [13] and Mendes et al. [11], according to which the effects of biostimulants can be interpreted more reliably by the combined evaluation of several functionally linked indicators than on the basis of isolated parameters.

It is noteworthy that the BiStep response was not consistently expressed as increased assimilation activity, but in *F. intermedia* and partly in *W. florida*, it was associated with differences in the relationship between Pn and E. This may indicate that the treatment influenced not only assimilation rates but also aspects of leaf-level physiological regulation, although this cannot be directly confirmed from the present dataset. According to Rathor et al. [21] and Arinaitwe et al. [32], the effect of humic- and fulvic acid-based products is often manifested in modifications at sensitive points of the regulation of plant physiological responses, rather than in changes of identical direction and magnitude in every taxon.

### 3.2. Linked Responses of the Photosynthetic Apparatus and Stomatal Regulation

This interpretation is supported by the significant taxon × treatment interaction detected for net photosynthesis and transpiration. In *W. florida*, changes in chlorophyll content under BiStep treatment occurred alongside differences in Pn and E, suggesting a coordinated response between pigment-related and gas-exchange parameters in this taxon. This pattern may indicate that the treatment influenced both stomatal functioning and the photosynthetic apparatus, although the lack of consistent statistical support across all parameters warrants cautious interpretation. More broadly, the higher Pn and lower E values observed in *F. intermedia* and partly in *W. florida* may be indicative of differences in internal water use efficiency, although direct confirmation of this would require the evaluation of more targeted gas exchange parameters.

Johnson et al. [18] describe this as one of the functional advantages of biostimulants, the coordinated improvement of photosynthetic functioning and physiological stability. In contrast, Jaros-Tsoj et al. [19] and Ferreira et al. [20] identified a similar tendency in systems where these two components shifted together in a favorable direction.

In *F. intermedia*, however, alongside the numerically higher net photosynthesis, chlorophyll content changed only slightly. This suggests that, in this taxon, the effect of the treatment may have been related less to modification of the pigment system and more to changes in gas exchange regulation. According to Siegwolf et al. [15], the relationship between photosynthetic functioning and gas exchange is not linear; therefore, higher assimilation performance does not necessarily require a change in pigments of the same magnitude.

This interpretation is further informed by the stomatal density and stomatal length data. Stomatal density increased across taxa, although the magnitude and statistical support of these changes differed. Based on Huang et al. [16] and Chen et al. [17], these traits together determine the morphological background of diffusive capacity; therefore, their functional meaning can only be interpreted together with gas exchange data. Based on the present results, morphological and physiological shifts showed greater concordance in *F. intermedia* and *W. florida* than in Viburnum, where the more restrained response profile may also indicate a different regulatory strategy.

### 3.3. Rhizosphere Enzyme Activities, Urban Applicability, and Novelty

Compared with leaf-level responses, rhizosphere enzyme activities provided a more differentiated but less uniform treatment picture. In the case of ACP, both treatment and interaction were significant, whereas ALP and GLUC did not show a uniform BiStep response. This suggests that the rhizosphere-level effect of the product in this experimental system did not appear as a uniform response extending to every enzyme examined, but rather showed a selective, taxon-dependent pattern. Differences in enzyme activity values compared with the previous *W. florida* study may be attributed to differences in experimental year, dataset structure, normalization, and the units used for expressing enzyme activity; therefore, the numerical values should not be directly compared without considering these methodological differences. This interpretation is particularly relevant for GLUC activity, where no significant treatment effects were detected. In *F. intermedia*, GLUC activity was numerically higher under BiStep treatment than in the control, although this difference was not statistically significant. This lack of significant change indicates that the treatment did not induce a measurable shift in β-glucosidase activity and highlights that rhizosphere responses were not necessarily aligned with leaf physiological responses. Overall, the results suggest that rhizosphere-level responses were enzyme-specific and taxon-dependent. Given the role of β-glucosidase in carbon cycling, the absence of a consistent response suggests that BiStep did not systematically alter this component of rhizosphere functioning within the conditions of this experiment. Wadduwage et al. [12] and Nicotra et al. [10] likewise emphasized that the soil biological consequences of biostimulants are strongly environment- and system-dependent, so leaf-level and rhizosphere-level responses are not necessarily parallel.

The practical significance of the study extends beyond container cultivation. The manuscript also emphasizes that the three taxa examined are important not only in nursery practice but also in urban green space applications. In this environment, fluctuations in water supply, restricted root zone, rapidly changing microclimate, and the need to reduce maintenance inputs make treatments particularly important when they may be associated with a more favorable leaf-level physiological condition. For urban plant species, Ankaya [30] emphasized that the integrated evaluation of leaf structural and functional traits is directly linked to sustainable plant use. The novelty of the present study lies in its taxon-comparative and multi-indicator approach. Unlike the previous *W. florida*-focused study, the present work evaluates BiStep responses across three ornamental shrub taxa and links gas exchange parameters with chlorophyll content, stomatal morphology, and rhizosphere enzyme activities. This integrated framework allows the treatment response to be interpreted not only as a change in individual physiological traits, but as a taxon-dependent functional response involving both leaf-level and rhizosphere-level indicators.

This integrated approach suggests that, from the perspective of urban ornamental shrub application as well, BiStep should be interpreted not as a universal input but as a tool to be considered in a taxon-specific manner. The most pronounced numerical leaf-level physiological response was observed in *F. intermedia*; however, this was not mirrored by a uniformly positive rhizosphere enzyme response, as no consistent or statistically significant change in β-glucosidase activity was detected under BiStep treatment in this taxon. The differing strength of leaf-level and rhizosphere-level responses also suggests that, in the system examined, the effect of BiStep was more clearly detectable at the level of plant physiological regulation, whereas rhizosphere biological consequences were more selective.

Overall, rhizosphere enzyme responses were not uniform across taxa or enzyme types, indicating that improved leaf-level physiological performance under BiStep treatment was not necessarily accompanied by consistent changes in rhizosphere biological activity. Therefore, the treatment effect should be interpreted as functionally selective rather than uniformly beneficial across physiological and rhizosphere-level indicators.

## 4. Materials and Methods

### 4.1. Plant Material and Experimental Design

The experiment was carried out in Zalaszentgyörgy, Hungary, approximately 7 km from Zalaegerszeg, under commercial container nursery conditions. The site is located in the Upper Zala Valley microregion, where the climate is moderately cool and moderately humid; annual sunshine duration is 1830–1950 h, mean annual temperature is 9.2–9.8 °C, annual precipitation is 700–800 mm, the prevailing wind direction is northerly, and average wind speed is below 3 m s^−1^ [35,36].

**Figure 5:**
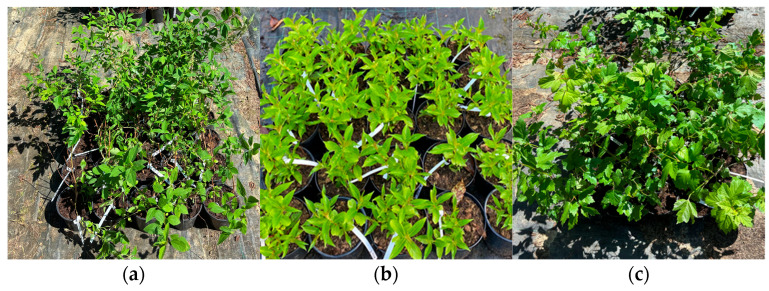
Representative images of the three ornamental shrub taxa included in the study: (**a**) *F. intermedia* ‘Beatrix Farrand’, (**b**) *W. florida* ‘Eva Rathke’, and (**c**) *V. opulus* ‘Roseum’. Images illustrate the general habit and foliage characteristics of the studied taxa.

Three deciduous ornamental shrub taxa were included in the study: *F. intermedia*, *W. florida*, and *V. opulus* (Figure 5). These taxa were selected because they represent well-known and widely used plant material in container nursery finished plant production while also being frequent, classic ornamental shrubs in urban green space plantings; consequently, the interpretation of treatment responses is relevant not only from a cultivation technology perspective, but also from planting and maintenance perspectives [37,38,39].

The plant material consisted of nursery-propagated, cutting-derived, one-year-old plants of uniform age and approximately identical size at the start of the experiment. Rooted cuttings were first grown in P9 pots according to nursery practice, then transplanted before experiment establishment into 1.5 L rigid-walled black plastic containers with an upper diameter of 13 cm, a lower diameter of 10 cm, and a height of 12 cm. The containers were filled with a mixture of 30% ground calcareous black peat and 70% ground white peat; the particle size of the substrate was 10–30 mm, and its EC value ranged between 2.0 and 4.0.

For the experiment, an approximately 20 m^2^ plot was designated in the container nursery. The flat surface was covered with UV-stable horticultural agrotextile with a 20 × 20 cm mesh pattern, which helped ensure uniform spacing. Two treatment levels were established for each taxon: untreated control and BiStep treatment, with 5 replicates, in a randomized block design. In the present study, the analysis was restricted to two treatment levels within each taxon: untreated control and BiStep treatment. This scope was chosen to evaluate the taxon-specific physiological and rhizosphere responses to the humic- and fulvic acid-based BiStep formulation under commercial container nursery conditions.

At experiment establishment, individuals of identical age, uniform development, and nearly identical size were selected within each taxon, and the study was conducted with independent biological replicates in a parallel arrangement.

### 4.2. Applied Biostimulant

Treatments were initiated after placement in the container nursery. The BiStep product used in this study is a humic- and fulvic acid-containing bioorganic plant conditioner. According to the official product authorization document, BiStep contains vermicompost extract, microorganisms, macro- and microelements, and water, with guaranteed nutrient contents including N, P_2_O_5_, K_2_O, MgO, Fe, Mn, Mo, Zn, and B [40]. The product is manufactured by UAB ALJARA (Vilnius, Lithuania). In the present study, the product was interpreted as a humic substance-containing biostimulant, expected to act by modifying the chemical conditions of the root zone and leaf-level physiological functioning [40]. Since the product examined is a complex formulation, the results can primarily be related to the functional pattern of the humic- and fulvic acid-based biostimulant treatment rather than to the independent mechanism of any single component.

### 4.3. Treatment and Application Protocol

The first treatment was applied simultaneously with containering in the first week of May 2025. Further treatments were carried out at two-week intervals during the growing season until shoot growth had ceased, which generally lasts until the end of August under cultivation conditions in the continental climate of Hungary, in the form of foliar spraying. BiStep was applied as a foliar spray at 0.5% concentration, in accordance with the official authorization document of the product [40]. The control plants received only water at the same times as the treatments, at 0.2 L per occasion. Timing and application schedule were identical for all three taxa in order to ensure that differences among treatments did not arise from differing phenological or technological schedules. Plants were irrigated through the nursery’s sprinkler system, using the same water rate and timing as in standard nursery practice; water supply was regulated by an automatic timer in two daily doses during the summer period.

### 4.4. Leaf-Level Gas Exchange Measurement

Leaf-level gas exchange parameters were determined using a portable photosynthesis measurement system operating on the principle of infrared gas analysis (LCi, ADC Scientific Ltd., Hoddesdon, UK). The system recorded photosynthetic rate and transpiration rate; in the present study, these were interpreted as net photosynthesis (Pn) and transpiration (E). Measurements were taken on fully developed, intact, assimilating leaves of both control and BiStep-treated plants within each taxon. Measurements were performed on a single day at two-hour intervals, selecting one sun leaf per plant. During sampling, leaf position and leaf surface integrity were checked uniformly to ensure that sampling-related differences did not bias interpretation.

### 4.5. Measurement of Chlorophyll Content

Chlorophyll content was determined according to the method of Arnon [41]. From the leaf samples, 150 mg fresh mass was weighed, then homogenized in chilled mortar and pestle in 80 V/V% acetone in the presence of quartz sand and Na_2_CO_3_. Samples were brought to a final volume of 10 mL and centrifuged, and the absorbance of the supernatant was measured spectrophotometrically at wavelengths of 663 nm, 644 nm, and 480 nm. Chlorophyll content was calculated using the standard formula and used as a leaf-level status indicator for interpreting physiological shifts associated with BiStep treatment. For gas exchange and chlorophyll measurements, *n* = 5 independent biological replicates were used per treatment group.

### 4.6. Determination of Stomatal Parameters

Stomatal parameters were determined from leaf impressions by microscopic image capture and image analysis. Sampling was carried out from fully developed, intact leaves, and both leaf position and the examined leaf zone were standardized within each taxon so that differences among treatments would genuinely reflect the relationship between leaf structure and treatment. Stomatal density was calculated from microscopic images by evaluating 10 leaves per treatment group and averaging two fields of view per leaf. Stomatal length was determined from the same impression-based images by morphometric analysis. Since, in the case of stomatal length, data were derived from several measurement points within a leaf, this variable was treated as a repeated observation during statistical analysis. The data for stomatal density and stomatal length were used to compare the control and BiStep treatments within each taxon. A Euromex bScope BS.1153-PLi biological microscope and a Euromex CMEX-5f camera (Euromex Microscopen BV, Arnhem, The Netherlands) were used for microscopic image capture.

### 4.7. Rhizosphere Sampling and Determination of Soil Enzyme Activities

Rhizosphere samples were taken from the root zones of the plants, and biological replicates were thoroughly homogenized within sample groups. Samples were stored chilled until analysis, and fresh material was used for enzyme activity determination. The activity of three enzymes was determined in the study: β-glucosidase (GLUC), acid phosphatase (ACP), and alkaline phosphatase (ALP).

β-glucosidase activity was determined according to the p-nitrophenol-based colorimetric method described by Eivazi and Tabatabai [42], using p-nitrophenyl-β-D-glucoside as substrate. After incubation, the reaction was stopped by adding pH 12 tris-hydroxymethyl-aminomethane (0.5 M) and 0.5 M CaCl_2_ solutions, and then the absorbance of the released PNP was determined spectrophotometrically at 410 nm. Blank samples were prepared in two ways: with a substrate-free blank control and with a suspension-free blank control; their absorbance values were subtracted from sample values. Activity was calculated on the basis of a standard curve.

Acid and alkaline phosphatase activities were determined according to the p-nitrophenyl phosphate method of Tabatabai and Bremner [43], using p-nitrophenyl phosphate as substrate. Samples were incubated at 30 °C in modified universal buffer, at pH 5 for ACP and pH 10 for ALP. After incubation, the reaction was stopped by adding 0.5 M NaOH, and the amount of PNP formed was determined spectrophotometrically. Enzyme assays were performed with four technical replicates per sample. Enzyme activities were expressed as µmol pNP g^−1^ h^−1^, representing molar pNP release rates calculated for the present experimental dataset.

### 4.8. Statistical Analysis

Data were analyzed using IBM SPSS Statistics for Windows, version 25.0 (IBM Corp., Armonk, NY, USA). For net photosynthesis (Pn), transpiration (E), and stomatal density, two-way analysis of variance was applied (GLM/UNIANOVA; Type III sums of squares), where taxon and treatment were included as fixed factors, and the taxon × treatment interaction was also tested. For soil enzyme activities, the same two-way model was run separately for each enzyme (ACP, ALP, GLUC), with taxon and treatment as fixed factors and including their interaction.

For the chlorophyll index, separate one-way analyses of variance were conducted within each taxon to compare treatments. Homogeneity of variances was checked with Levene’s test. When the assumption of homogeneity of variance was not met, Welch’s robust analysis of variance was applied.

The stomatal length dataset was evaluated using a linear mixed model because repeated observations were obtained from several measurement points within a leaf. Taxon, treatment, and the taxon × treatment interaction were included as fixed effects, while leaf identity was included as a random intercept to account for within-leaf dependence. Pairwise comparisons were performed based on estimated marginal means, using Bonferroni correction for GLM/UNIANOVA models and Sidak adjustment for the linear mixed model where applicable. Effect size was given as partial eta squared (ηp^2^), and the significance level was α = 0.05. Data are presented as mean ± standard error (SE).

## 5. Conclusions

Biostimulant treatment may modify leaf-level physiological responses in containerized ornamental shrub production, but the magnitude and functional significance of these effects are clearly taxon-dependent and are not consistently reflected across all rhizosphere enzyme indicators. This is important in practical terms because the improved physiological state achieved during containerized finished plant production may contribute to improved post-planting performance and stress tolerance, although this requires direct confirmation under field conditions.

The results showed that the treatment effect does not follow a uniform pattern across taxa; therefore, practical evaluation and recommendation of biostimulants cannot be carried out on a taxon-independent basis. The data obtained indicate that the combined examination of leaf-level gas exchange, chlorophyll content, stomatal morphological traits, and rhizosphere enzyme activities may provide a suitable framework for a more precise assessment of treatment responses, because together these parameters sensitively indicate not only short-term physiological status but also the stability of plant functioning. It also follows from this that the effectiveness of biostimulants should be interpreted in the context of nursery finished plant production and subsequent urban use, especially in environments where restricted root zone, periodic water deficit, increased heat load, and the need to reduce maintenance interventions simultaneously shape plant performance. The novelty of the study lies in evaluating treatment effects within an integrated system of several functionally related physiological and rhizosphere-level indicators. This approach may lay the foundation for more targeted, taxon-specific application of biostimulants and strengthen the relationship between nursery treatments and post-planting performance in urban settings. Further studies should therefore examine the extent to which the physiological advantages detectable during container production persist after planting out, and how they are modified under different site, irrigation, and maintenance conditions.

## Data Availability

Available on request.

## Abbreviations

The following abbreviations are used in this manuscript:

*F. intermedia*	*Forsythia* × *intermedia* ‘Beatrix Farrand’
*W. florida*	*Weigela florida* ‘Eva Rathke’
*V. opulus*	*Viburnum opulus* ‘Roseum’
ACP	acid phosphatase
ALP	alkaline phosphatase
GLUC	β-glucosidase
